# Editorial Notes

**Published:** 1923-07

**Authors:** 


### Editorial Notes.

**Professor E. Fawcett, F.R.S.**

THE election of Professor Fawcett to the Fellowship of the Royal Society has given great satisfaction to all his colleagues and to many generations of Bristol Medical Students.

Professor Fawcett came to Bristol in 1893 to fill the newly founded Chair of Anatomy in University College. In 1905 he succeeded Dr. Markham Skerritt as Dean of the Faculty of Medicine. He has held both these offices with notable distinction and much advantage to the University. His researches in Anatomy, more particularly those dealing with the comparative morphology of the chondro-cranium won world-wide recognition, so that it was no surprise to Bristol that he should be elected F.R.S. As a teacher of Anatomy we have long since realised that he stood in the front rank. His prowess in the cricket-field, his knowledge of ecclesiastical architecture, and his wide acquaintance with archæology and anthropology bear witness to the universality of his interests.

We offer Professor Fawcett our heartiest congratulations that the Royal Society should have crowned his labours with such a well-earned honour.

**Resignation of Miss A. B. Baillie, R.R.C.**

IT is with great regret that we have to record the resignation of Miss Baillie from the Matronship of the Royal Infirmary.

Miss Baillie received her nursing training under Miss Luckës at the London Hospital, where she became Sister of the Surgical and Accident Wards, and later Matron's Office Assistant. In 1896 she was appointed

Matron of the Hospital of St. Cross at Rugby. In 1898 she succeeded Miss Smith as Matron of the Bristol Royal Infirmary. Her work there is too well known to need detailing. Under her guidance the nursing department has made marvellous progress. More than twenty of her nurses at present occupy matron's posts.

As soon as she assumed office she materially increased the number and the efficiency of the nursing staff. The New Nurses' Home and its magnificent garden owe their existence to her energy. She started a massage school for nurses at the Infirmary in 1904, and a preliminary training school for nurses in 1908, the first to be opened in the provinces. One great innovation which Miss Baillie was the first Matron in the country to practise was that of giving each nurse a whole day off duty every week. The present successful midwifery school for nurses is another proof of her organising ability—and the new lying-in wards which were opened after the war were in large part due to her endeavours. On the formation of the Territorial Force Nursing Service, Miss Baillie was made a Principal Matron. Her work in this capacity during the war was beyond praise ; and when she received the order of the Royal Red Cross (first class) in 1915, it was universally felt that no one had more richly deserved the honour. She had not only been responsible for the staffing of the 2nd Southern General Hospital with nurses, but had also contributed largely to the organisation of many subsidiary Red Cross Hospitals in the Southern Command and had arranged for the supply of nurses to these hospitals.

For the past three years Miss Baillie has been a member of the Army Nursing Council, and she is also on the Council of the College of Nurses.

In 1922 the Committee of the Bristol Royal Infirmary in recognition of the great part played by her in the development of the maternity department named one of the wards the " A. B. Baillie " Ward.

Miss Baillie is now leaving the Infirmary to become the first Matron of the St. Monica's Home for incurables, founded by the late Mr. H. H. Wills.

She takes with her to her new work the good wishes of all who have worked with her, and a feeling of confidence that her unabated energy and organising capacity will ensure the success of St. Monica's Home.

**Colston Research Society.**

THE Annual Dinner of the Colston Research Society was held at the Victoria Rooms, Clifton, on Friday, June 1st, under the presidency of Mr. Claude Fry, and was well attended. The Toast List was a short one, the principal speeches being those of Sir W. M. Petrie and Sir Richard Gregory, the two guests, who replied to the Toast of their health proposed by the Vice-Chancellor. The Presidential Collection amounted to £669 8s. 6d., and it was satisfactory to learn that the Colston Research Fellowship Scheme initiated last year has been so successful that five out of the six firms then donating Fellowships have renewed them for a further period, and two other firms have taken advantage of the Scheme, making seven Fellowships in all. The Society is making headway and getting increased support from the Commercial Community, between whom and the University it is helping to promote a closer liaison.

**The British Journal of Surgery.**

THE *British Journal of Surgery* enters with the July number just published upon the eleventh year of issue. The long continuance of the Great War sadly handicapped its production by doubling, and at one time actually trebling, its cost. The wonderful way in which members of the Medical Profession generally continued to support their new Journal during those trying years will always remain a happy memory with the proprietors. It is therefore with pride as well as pleasure that they find themselves able, with the commencement of the present volume, to return to practically pre-war conditions. The type is improved, the pages are increased to 200 per number, and no opportunity is being missed to enhance still further the value of the contents and the way in which it is presented. This publication not only represents the highest attainments of British Surgery, but also exhibits the best that is obtainable in artistic medical journalism.

